# Semi-quantitative analysis of cannabinoids in hemp (*Cannabis sativa* L.) using gas chromatography coupled to mass spectrometry

**DOI:** 10.1186/s42238-022-00161-w

**Published:** 2022-09-22

**Authors:** Luca De Prato, Matthew Timmins, Omid Ansari, Katinka X. Ruthrof, Giles E. St. J. Hardy, John Howieson, Graham O’Hara

**Affiliations:** 1 Food Future Institute, Murdoch University, Murdoch, Western Australia 6150 Australia; 2 Medicann Health Aust Pty Ltd., South Perth, Western Australia 6151 Australia; 3 Solarbike, Fremantle, Western Australia 6160 Australia; 4 Ecofibre Ltd., PO Box 108, Virginia, Queensland 4014 Australia; 5 Ananda Food Pty Ltd., Beresfield, New South Wales 2322 Australia; 6 Department of Biodiversity, Conservation and Attractions, Kensington, Western Australia 6151 Australia

**Keywords:** Cannabinoids, GC-MS, Extraction, Trichomes, Low-abundance, Industrial hemp

## Abstract

**Background:**

Hemp (*Cannabis sativa* L.) is a producer of cannabinoids. These organic compounds are of increasing interest due to their potential applications in the medicinal field. Advances in analytical methods of identifying and quantifying these molecules are needed.

**Method:**

This study describes a new method of cannabinoid separation from plant material using gas chromatography-mass spectrometry (GC-MS) as the analytical tool to detect low abundance cannabinoids that will likely have implications for future therapeutical treatments. A novel approach was adopted to separate trichomes from plant material to analyse cannabinoids of low abundance not observed in raw plant extract. Required plant sample used for analysis was greatly reduced compared to other methods. Derivatisation method was simplified and deconvolution software was utilised to recognise unknown cannabinoid compounds of low abundance.

**Results:**

The method produces well-separated spectra and allows the detection of major and minor cannabinoids. Ten cannabinoids that had available standards could be identified and quantified and numerous unidentified cannabinoids or pathway intermediates based on GC-MS spectra similarities could be extracted and analysed simultaneously with this method.

**Conclusions:**

This is a rapid novel extraction and analytical method from plant material that can identify major and minor cannabinoids using a simple technique. The method will be of use to future researchers seeking to study the multitude of cannabinoids whose values are currently not understood.

## Introduction

Over the last few years, a renewed interest in *Cannabis sativa* and its products has occurred worldwide due to the easing of legislation (Cox 2018; Mead 2017). Several countries, such as Canada, Germany, Thailand and Australia, have legalised *Cannabis* products for medicinal purposes (Cox 2018; Guiney 2017; Kumar et al. 2019). The psychoactive and therapeutic properties used for medicinal products are related to chemical compounds produced by *C. sativa* called cannabinoids. The more recognised active ingredients are the psychoactive delta-9-tetrahydrocannabinol (Δ^9^-THC or THC) and the therapeutic cannabidiol (CBD) (Small et al. 2003). *Cannabis sativa* is grouped into three different chemotypes based on the THC and CBD ratio and concentration: (1) a drug type with low CBD/THC (chemotype I); 2) a less common type with CBD/THC ranging between 0.5 and 3.0 (chemotype II); 3) and a no-drug type with high CBD/THC > 3.0 (chemotype III) more commonly called hemp (Pacifico et al. 2006; Small and Beckstead 1973). In Western Australia, hemp is defined as having a THC concentration of < 1% by dry weight (Allsop and Hall 2017; Department of Primary Industries and Regional Development 2020). However, hemp can contain numerous and substantial concentrations of cannabinoids other than THC and other types of molecules with pharmaceutical value, such as CBD and minor cannabinoids, terpenoids, flavonoids, and phenols (Calzolari et al. 2017; Citti et al. 2018). Recent studies are examining potential new pharmaceutical properties related to the idea of the ‘entourage effect’ through doses of a combination of cannabinoids (Booth et al. 2017; Russo 2018).

Cannabinoids are a group of more than 130 recognised C21 meroterpenoles or terpenophenolic compounds produced from fatty acids and isoprenoid precursors unique to the *Cannabis* genus (Carvalho et al. 2017; Citti et al. 2019; Hanus et al. 2016). Their roles within the plant are not fully understood (Pacifico et al. 2008). Twelve major cannabinoids are recognised to have therapeutic properties: Δ^9^-tetrahydrocannabivarin (THCV), cannabidiol (CBD), cannabigerol (CBG), Δ^8^-tetrahydrocannabinol (Δ^8^-THC), cannabichromene (CBC), cannabinol (CBN), cannabidiolic acid (CBDA), Δ^9^-tetrahydrocannabinolic acid (THCA), tetrahydrocannabivarin acid (THCVA), cannabigerolic acid (CBGA), cannabidivarin (CBDV), and Δ^9^-tetrahydrocannabinol (Δ^9^-THC) (Cardenia et al. 2018; Leghissa et al. 2018b). Additionally, a novel and important cannabinoid of low abundance, Δ^9^-tetrahydrocannabiphorol (Δ^9^-THCP), was recently discovered (Citti et al. 2019), which could help partly explain the psychoactive effect of *Cannabis.* Plant breeding can enhance the production of selected cannabinoids to produce a high-value crop (Small and Marcus 2003), so the ability to detect cannabinoids of low abundance or of currently unknown therapeutic value needs to be improved and simplified to give more detailed information about different chemotypes.

**Fig. 1:**
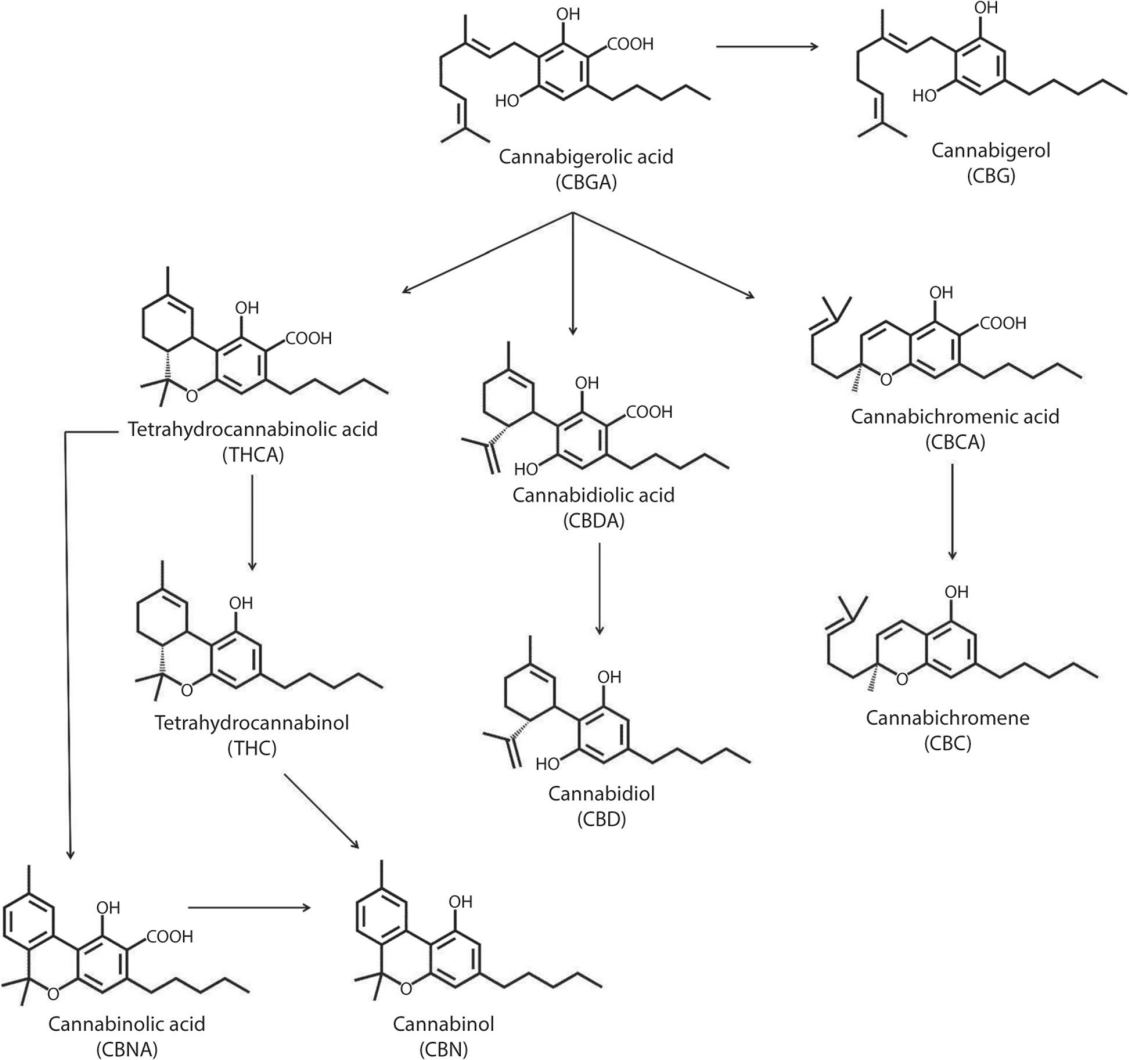
Structures and biosynthetic pathway of the main cannabinoids in *Cannabis sativa* (Aizpurua-Olaizola et al. 2016).

Understanding conditions that change the concentration of secondary metabolites and the plant biosynthesis process is of interest because such knowledge could improve the production of targeted cannabinoids. *Cannabis sativa* is known to produce carboxylated versions of cannabinoids; cannabidiol acid (CBDA) is the precursor of cannabidiol (CBD), as discovered by Schulz and Haffner in the 1960s (Cardenia et al. 2018; Hanus et al. 2016). Therefore, those cannabinoids need to undergo spontaneous decarboxylation through air drying or heat treatment to become an active ingredient for the endocannabinoid system (Aizpurua-Olaizola et al. 2016). It is possible to find cannabinoids and terpenes in various parts of the plant, both male and female, in the vegetative and flowering stages; however, the higher concentrations are found in resin secreted by epidermal glands called trichomes (Turner et al. 1980). Trichomes are abundant mostly around the female flower (Booth et al. 2017). However, the accumulation of cannabinoids is a process that changes over the growth cycle of *C. sativa* (Hanus and Dostálová 1994; Pacifico et al. 2008). For example, increased physiological age of hemp leaves resulted in an increase followed by a decrease in THC concentration (Andre et al. 2016; Bócsa et al. 1997; Khajuria et al. 2020). Furthermore, Δ^9^-THC is produced by its precursor THCA, and it oxidases into CBN (Fig. 1) (Leghissa et al. 2018b). Routine testing uses mature female flowers when the cannabinoid production of THC is roughly at its peak when pistillate stigmas turn dark orange on mature flowers (Small et al. 2003). However, the cannabinoid profile evolves during the plant life cycle, even though the phenotypical cannabinoid ratio (THCA/CBDA) tends to remain equal during the plant life cycle (Aizpurua-Olaizola et al. 2016). This is an essential aspect to consider if forensic studies have to identify whether the plant is a drug chemotype or not, and when the plant material should be tested.

Comprehension of the biomedical applications and biosynthesis process of cannabinoids in the plant (Fig. 1) are fundamental to understand the range of analytical methods that can be used to identify and quantify the number and quantity of cannabinoids within a sample. Due to the high variability of each cannabinoid content in *C. sativa* plants (Aizpurua-Olaizola et al. 2016), sample timing and preservation, extraction, and analytical instrument tuning are critical. Over the last few years, many studies have undertaken method development for cannabinoid detection (Elkins et al. 2019; Leghissa et al. 2018b; Nahar et al. 2020). Mostly, methanol (CH_3_OH) and ethanol (C_2_H_5_OH) are used for extraction, followed by analytical instrument methods for determination and quantification (Nahar et al. 2020). The most common analytical methods use high-performance liquid chromatography (HPLC) with an ultraviolet detector (HPLC-UV), or gas chromatography (GC) with electron ionisation (EI) coupled to mass spectrometry (MS) or a flame ionisation detector (FID) (Brighenti et al. 2017; Cardenia et al. 2018; Citti et al. 2018). The most used instrument for commercial testing is HPLC, as it is cost-effective, has a faster sample preparation and process than other instruments; however, GC-MS has a higher sensitivity for detection of chemical molecules and, also, due to the mass spectrometer, can be utilised for identification using compound libraries and, with the addition of the EI and FID, can detect volatile chemical compounds (Leghissa et al. 2018b). Considerations have been made about the hot injection of a GC-MS given the fact that the cannabinoids are decarboxylated by heat (Leghissa et al. 2018b). For this reason, derivatisation (silylation or methylation/esterification) must be undertaken pre-analysis (Fodor and Molnár-Perl 2017). The process stabilises the cannabinoid molecules attaching a methylic group to them, making the compound stable and easily detectable. In this process, several procedures have been reported with the utilisation of N-methyl-N-(trimethylsilyl)trifluoroacetamide (MSTFA), while other researchers have used the combination of N,O-bis(trimethylsilyl)trifluoroacetamide (BSTFA), or MSTFA (Fodor and Molnár-Perl 2017; Leghissa et al. 2018a; Nahar et al. 2020). In general, the use of GC-MS in a routine analysis was usually not considered, given the long run time. However, the development of Fast GC-MS methodology has made it commercially competitive (Cardenia et al. 2018). In forensic research for drug analysis, a Fast GC-MS method has been applied by Byrska and Zuba (2009), reducing the column length, increasing the carrier gas flow, and raising the MS oven temperature. A more recent study by Cardenia et al. (2018) tested a Fast GC-MS showing its potential to detect cannabinoids in hemp, the low THC varieties of *C. sativa*. However, in their extraction method, Cardenia et al. (2018) used large amounts of plant sample (around 25 g) and chemicals for extraction. In contrast to HPLC, a GC coupled with FID can be used to detect terpenes, the fragrant molecules that *C. sativa* produces, which are recognised to have medicinal value (Ibrahim et al. 2019). Since the discovery of the first cannabinoid (THC) (Mechoulam and Gaoni 1965), more than 130 cannabinoids have been identified (Carvalho et al. 2017; Citti et al. 2018, 2019; Leghissa et al. 2018b). The MS is a unique tool for untargeted and low abundance compounds, which could present therapeutic properties (Capriotti et al. 2021). However, most of these can be extracted in only very low amounts, and the commercial availability of quality synthesised cannabinoid standards with an extended range is a problem for identification (Carvalho et al. 2017).

This study describes the improvements made on earlier methods of cannabinoid detection, simplifies the identification process, and increases the detection of some minor cannabinoids, which usually occur at a lower concentration in the no drug chemotype (III) of *C. sativa*. Fast GC-MS has been previously used for narcotic drugs and psychotropic substances (Byrska and Zuba 2009) and cannabinoids with good results (Cardenia et al. 2018). A GC-MS was utilised, but purer cannabinoid extractions were also explored to improve the detection of low abundance cannabinoids in hemp. Also, to speed up and simplify extraction methods, a comparison of derivatisation reagents was investigated. In summary, this study aimed to improve the method for cannabinoid testing through an easier and leaner extraction method and higher detection of the low abundance metabolites on a Fast GC-MS, with a focus on hemp.

## Materials and methods

### Standard and reagents

Hexane, methanol, HPLC grade water, ethanol, 5-alpha-cholestane and N-methyl-N-(trimethylsilyl) trifluoroacetamide with 1% trimethylchlorosilane (MSTFA) were purchased from Sigma-Aldrich/Merk (Bayswater, VIC, Australia). Methanol was used as a solvent to extract cannabinoids from plant material. The derivatisation agents utilised were N-methyl-N-(trimethylsilyl)trifluoroacetamide (MSTFA) and N,O-bis(trimethylsilyl)trifluoroacetamide (BSTFA) that were purchased from Sigma-Aldrich/Merk (Bayswater, VIC, Australia). An Agilent 7890BGC+5977E MSD (Agilent Technologies Australia, Mulgrave, VIC, Australia) was utilised for the detection of the plant material compounds.

Certified cannabinoid standard were produced by Cayman Chemical (Ann Arbor, Michigan 48108 USA) and acquired by Cannalab (Perth, Western Australia). The cannabinoid standard (product code: 21305) contained tetrahydrocannabinolic acid (THCA), Δ^9^-tetrahydrocannabinol (Δ^9^-THC), cannabidiol (CBD), cannabidiolic acid (CBDA), cannabinol (CNB), cannabigerol (CBG), cannabigerolic acid (CBGA), cannabichromene (CBC), cannabidivarin (CBDV), and Δ^8^-tetrahydrocannabinol (Δ^8^-THC) at 250 μg/mL of each compound. However, synthetic standards for the qualification of the main cannabinoids (CBDV, CBD, CBC, CBDA, THCA, Δ^9^-THC, Δ^8^-THC, THCV, CBG, CBN, THCVA, and CBGA) other than the top 12 are not commercially available yet. Therefore, the identification of the low abundance and unidentified compounds was carried out with deconvolution software.

### Separation of trichomes from plant material

**Fig. 2:**
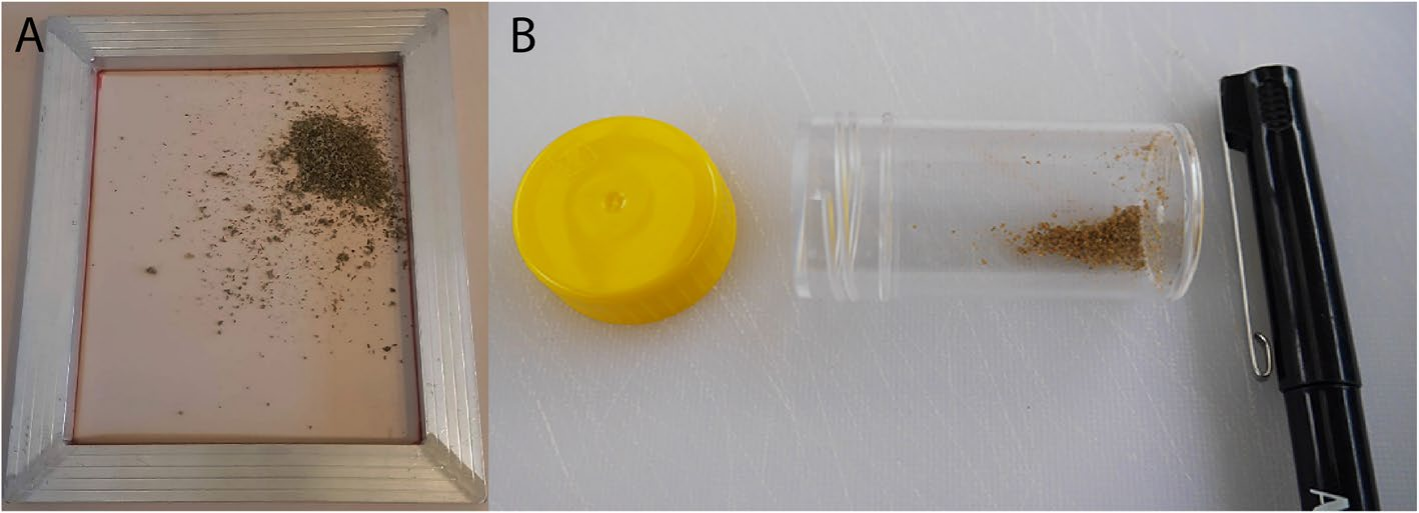
**A** Obtaining cannabinoid-rich trichomes from *Cannabis sativa* is achieved via gentle shaking of frozen plant material over a 40-μm nylon mesh pulled tightly over a frame (33 cm wide × 38 cm long × 2.5 cm deep). **B** Trichome-rich material post-filtration in a 25 ml vial. Pen and lid positioned for scale

Air-dried inflorescences from a mixture of two different *C. sativa* accessions (a chemotype I and a chemotype III), were collected and mixed to create a base with a wide range of cannabinoids. Glandular material that accumulates in the trichomes of *C. sativa* is highly concentrated in cannabinoids and becomes brittle at low temperatures. Frozen trichomes readily separate from the plant material and can be purified by filtration. Plant material was frozen for 30 min at – 4 ^o^C. The dried and frozen plant material was then sieved through a 40-μm nylon mesh filter (Swiss Screens, Perth, Western Australia) (Fig. 2A). The trichomes fall through the filter, leaving the majority of plant material behind. This trichome-rich filtrate (Fig. 2B) contains a high concentration of cannabinoids. Between 3 and 4 mg of trichome filtrate is used for the extraction of cannabinoids.

### Extraction of cannabinoids from trichomes and plant material

Dried plant material was ground using an electric spice grinder (the Coffee and Spice, model: BCG200BSS, Breville Group, Sydney NSW, Australia). For each sample, 10.5 ± 0.3 mg of dried plant material was weighed into 2 ml micro-centrifuge tubes. If using only trichome-rich extract, then 3.5 ± 0.1 mg of the material was used. To extract cannabinoids, 500 μl of methanol with 20 μg of 5-alpha-cholestane (internal standard) was added to each sample. This was followed by the addition of 500 μl of n-Hexane and 500 μl of water. Samples were agitated on a heat block at 40 °C for 5 min using an Eppendorf ThermoMixer C (Hamburg, Germany, Product nr. 232000083). Samples were then centrifuged at 28,000×*g* for 5 min to achieve phase separation with a ThermoFisher Sorvall ST1 Plus (Waltham, Massachusetts, USA, Product nr. 75009740). One hundred microliters of the upper organic layer (n-Hexane) was transferred to a GC-MS vial insert and allowed to dry under a gentle nitrogen stream at room temperature.

### Derivatisation and analysis of cannabinoids

A 90 μl of MSTFA was added directly to the GC-MS vial insert containing extracted and dried cannabinoid sample. Silylation was performed at 60 ^o^C for 25 min. One microliters of the derivatised sample was injected into an Agilent 7890BGC+5977E MSD under splitless mode. The injection inlet was set at 300 °C, and the GC purge valve was set to be switched on at 1 min after injection. An Agilent HP-5MS 15 m × 250 μm, 0.25 μm column was used for separation. Ultra-High Purity helium high flow was used as the carrier gas at a constant flow rate of 1.2 ml min^−1^. The initial oven temperature was held at 80 °C for 0.5 min, then ramped up to 250 °C at 40 °C min^−1^ and then to 300 °C at 10 °C min^−1^. The mass selective detector (MSD) transfer line, ion source, and quad-pole temperatures were 280, 280, and 150 °C, respectively.

### Identification and analysis

**Fig. 3:**
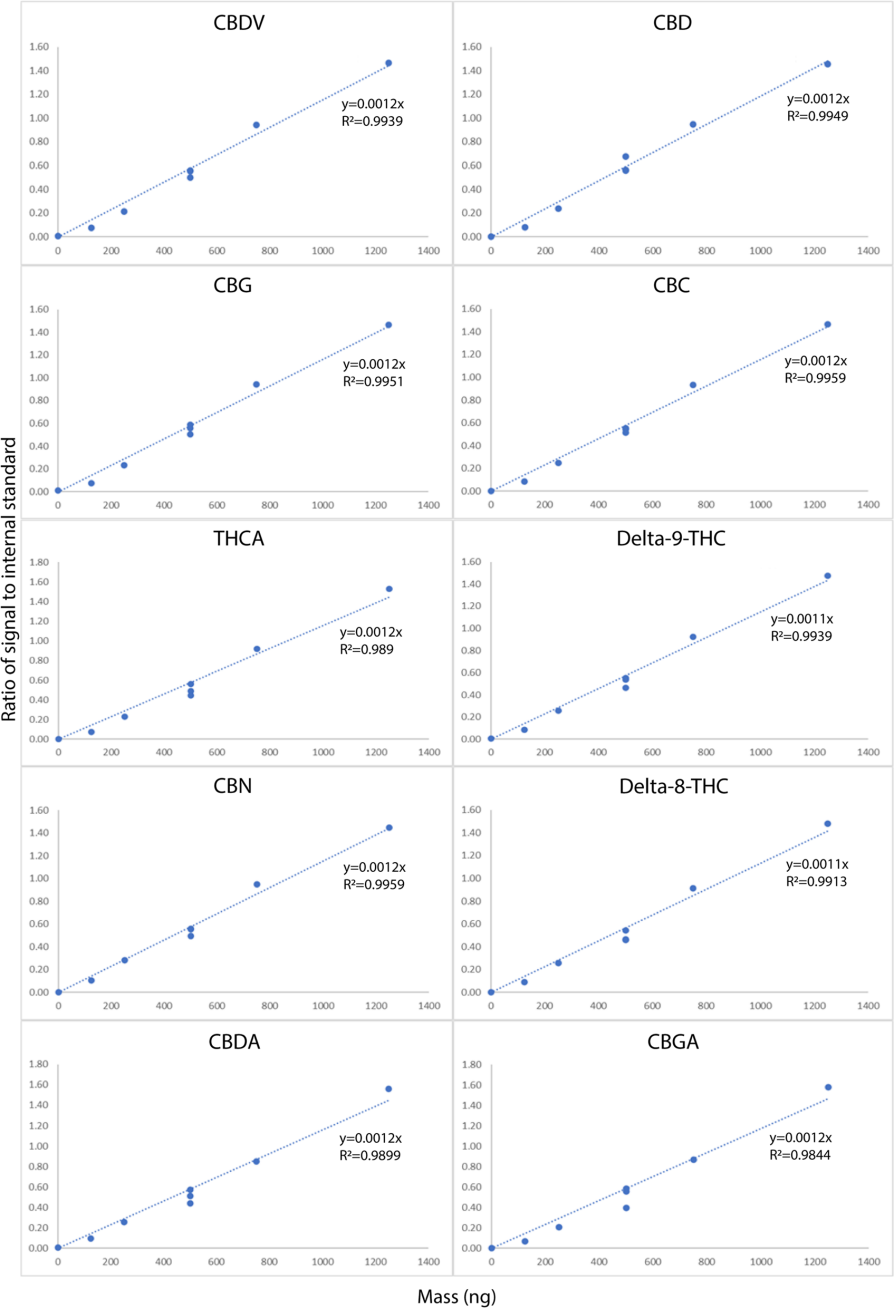
Linear range of ten cannabinoids from the Cayman Chemical Cannabinoid standard Mixture 10 (product code: 21305) on six increasing concentrations (0; 125 ng/mL; 250 ng/mL; 500 ng/mL; 750 ng/mL; 1250 ng/mL) injected on the GC-MS calculated by the ratio of signal of internal standard on increasing mass (ng) for each cannabinoid (CBDV = cannabidivarin; CBD = cannabidiol; CBG = cannabigerol; CBC = cannabichromene; THCA = tetrahydrocannabinolic acid; Delta-9-THC = Δ^9^-tetrahydrocannabinol; CBN = cannabinol; Delta-8-THC = Δ^8^-tetrahydrocannabinol; CBDA = cannabidiolic acid; CBGA = cannabigerolic acid)

Cannabinoids were identified by comparison to purchased standards and to spectra from publications (Cardenia et al. 2018; Feyerherm and Macherone 2015) and confirmed with the National Institute of Standards and Technology database (NIST 2018) (Shen et al. 2020). Library construction and quantitation of known compounds were performed by Agilent Mass Hunter Quantitative Analysis Software (ver. 6.0). Calibration curves were created using concentrations of available standards between 0 and 1250 ng/L on column using the above-mentioned extraction and derivatisation techniques (Fig. 3). Agilent GC-MS Mass Hunter Software was used for data acquisition and analysis. Samples were randomised and analysed in two independent analytical runs. The mass spectra of unknown compounds were deconvoluted through AMDIS_32 (ver. 2.64), and peaks with increased probability of representing cannabinoids or intermediates of synthetic cannabinoid pathways were identified based on their mass spectral quantification and qualification ions being present in over three cannabinoid standards used in this study. Peak detection, deconvolution, filtering, scaling, integration, and quantitation were conducted in the Mass Hunter Quantitative Analysis for GC-MS Software (Ver. 7.045.7). The method of cannabinoid extraction and analysis developed in this study enabled the quantification of cannabinoids of low abundance that will be targeted for future medicinal uses.

### Method validation

Cannabinoid quantification was based on the use of the cannabinoid standard and the accuracy was adjusted through the response linearity evaluated for each cannabinoid. The calibration curve created were calculated by injecting six increasing concentrations of cannabinoid standard (0; 125 ng/mL; 250 ng/mL; 500 ng/mL; 750 ng/mL; 1250 ng/mL) on the GC-MS (Fig. 3) column. The limit of detections (LOD) and quantification (LOQ) were calculated by the signal to noise ratio on mass (ng). Intraday and interday precision of the GC-MS instrument (RSD) were calculated with three technical repetitions of the mid cannabinoid standard concentration (500 ng/mL) (Table 1). Also, technical and pool sample repetitions (min *n* = 3) for each batch were injected every seven samples to test the continuous functionality and accuracy of the instrument.

**Table 1:** Analytical parameters of a GC-MS method for RSD (intraday and interday accuracy of repeated injections; %), LOD (limit of detection) and LOQ (limit of quantification) for cannabinoid internal standard (Cannabinoid Mixture 10; Cayman Chemical, Ann Arbor, MI, USA) injected three times at a concentration of 500 ng/mL

	**CBDV (mg/kg)**	**CBD (mg/kg)**	**CBG (mg/kg)**	**CBC (mg/kg)**	**Δ8-THC (mg/kg)**
**Repeat-1 @ 500 ng**	0.55	0.56	0.56	0.55	0.55
**Repeat-2 @ 500 ng**	0.56	0.56	0.59	0.54	0.47
**Repeat-3 @ 500 ng**	0.50	0.68	0.51	0.51	0.46
**Average**	0.54	0.60	0.55	0.54	0.49
**2xSD**	0.07	0.13	0.08	0.04	0.10
**RSD**	6%	11%	7%	4%	10%
**LOD**^**b**^	82.31	166.40	100.93	50.19	118.90
**LOQ**^**b**^	274.36	554.65	336.42	167.30	396.32
	**Δ9-THC (mg/kg)**	**CBDA (mg/kg)**	**CBN (mg/kg)**	**CBGA (mg/kg)**	**THCA (mg/kg)**
**Repeat-1 @ 500 ng**	0.54	0.44	0.56	0.40^a^	0.45
**Repeat-2 @ 500 ng**	0.55	0.57	0.55	0.59	0.56
**Repeat-3 @ 500 ng**	0.46	0.51	0.49	0.56	0.49
**Average**	0.52	0.51	0.53	0.57	0.50
**2** × **SD**	0.10	0.13	0.07	0.05	0.12
**RSD**	9%	13%	7%	4%	12%
**LOD**^**b**^	122.37	164.20	88.90	56.49	144.68
**LOQ**^**b**^	407.91	547.33	296.34	188.30	482.28

^a^0.40 ratio is an outliner

^b^LOD/LOQ are based on 3 × SD/10 × SD

## Results

**Fig. 4:**
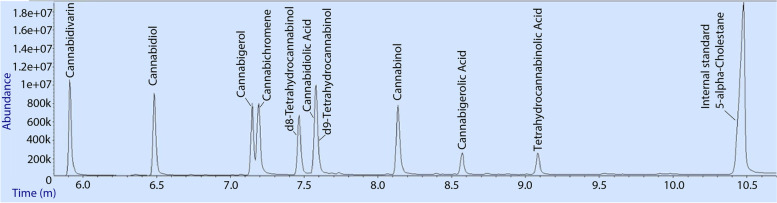
A total ion current chromatogram showing the elution of the ten cannabinoids in the Cayman Chemical Cannabinoid standard Mixture 10 (product code: 21305), 70 ng on column and 5-alpha-cholestane as internal standard. Cannabidiol acid and Δ^9^-tetrahydrocannabinol coeluted in one peak so deconvolution is required for quantification

The developed analytical method was able to separate nine of the 10 cannabinoid standard mixture based on retention time alone (Fig. 4). Two compounds, CBDA and Δ^9^-THC coeluted with this analytical method and the power of the mass spectrometer was required to distinguish them. Retention times, quantification, and qualification ions suitable for the 10 cannabinoid standard compounds are shown in Table 2.

**Table 2:** Compounds, retention time (min), quantifier and qualifier ions of Cayman Chemical Cannabinoid standard Mixture 10 (product code: 21305). Derivatisation groups as per Cardenia et al. (2018)

Compound	Retention time (min)	Quantifier ion	Qualifier ions	Derivatisation group(s)
Cannabidivarin	5.91	362	309, 273	CBDV-1TMS
Cannabidiol	6.48	390	337, 301	CBD-2TMS
Cannabigerol	7.15	337	391, 460	CBG-2TMS
Cannabichromene	7.19	303	246, 371	CBC-1TMS
Δ^8^-tetrahydrocannabinol	7.47	386	303, 330	Δ8-THC-1TMS
Cannabidiolic acid	7.56	491	559, 453	CBDA-3TMS
Δ^9^-tetrahydrocannabinol	7.58	371	386, 315	Δ9-THC-1TMS
Cannabinol	8.14	367	382, 310	CBN-1TMS
Cannabigerolic acid	8.57	561	417, 453	CBGA-3TMS
Tetrahydrocannabinolic acid	9.08	487	488, 502	THCA-2TMS
^a^5-alpha-cholestane	10.48	217	357, 372	–

^a^Internal standard

**Fig. 5:**
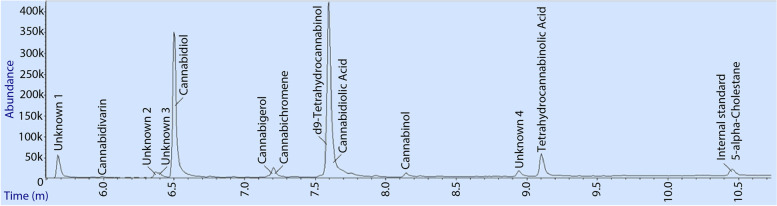
Total ion current chromatogram from a 10.5 ± 0.3 mg hemp plant sample of *Cannabis sativa* showing elution of eight cannabinoids identified from standards and elution of four unknown compounds likely to represent unidentified cannabinoids

Identification and quantification of cannabinoids from an extract from 10.5 ± 0.3 mg of hemp apical plant material allowed identification and quantification of 8 of the 10 cannabinoids from the cannabinoid standard mixture (Fig. 5). It was also possible to quantify four unknown compounds that likely represented cannabinoids as either end-products or intermediates of a cannabinoid synthetic pathway based on the presence of unique spectral ions similar to those of known cannabinoids. The increased complexity of the plant metabolic matrix resulted in a partial coelution of CBG and CBC on one peak, and Δ^9^-THC and CBDA on another. These compounds could be identified and quantified based on their unique mass spectral profile (Fig. 5; Table 3).

**Table 3:** Compound, retention time (min), quantification and qualifying ions of cannabinoids, and unknown compounds from 10.5 ± 0.3 mg of the mixed inflorescences plant material with a *Cannabis sativa* accession (chemotype III)

Compound	Retention time (min)	Quantifier ion	Qualifier ions	Sample 1 (mg/kg)	Sample 2 (mg/kg	Sample 3 (mg/kg)
Unknown 1	5.68	313	117, 129	–	–	–
Cannabidivarin	5.91	362	309, 273	2.6	2.6	1.6
Unknown 2	6.37	341	129, 145	–	–	–
Unknown 3	6.40	337	262, 129	–	–	–
Cannabidiol	6.48	390	337, 301	301.5	196.0	186.3
Cannabigerol	7.17	337	391, 460	6.5	4.5	4.3
Cannabichromene	7.19	303	246, 371	42.3	28.2	26.5
Δ^8^-tetrahydrocannabinol	7.47 (not detected)	386	303, 330	1782.0	1096.3	996.3
Δ^9^-tetrahydrocannabinol	7.59	371	386, 315	1730.9	1079.9	972.3
Cannabidiolic acid	7.60	491	559, 453	1583.8	1095.3	987.6
Cannabinol	8.14	367	382, 310	22.1	11.9	13.7
Cannabigerolic acid	8.6 (trace)	561	417, 453	16.3	15.1	11.2
Unknown 4	8.94	419	487, 257	–	–	–
Tetrahydrocannabinolic acid	9.08	487	488, 502	9205.7	7354.1	6858.3
^a^5-alpha-cholestane	10.46	217	357, 372	–	–	–

^a^Internal standard

**Fig. 6:**
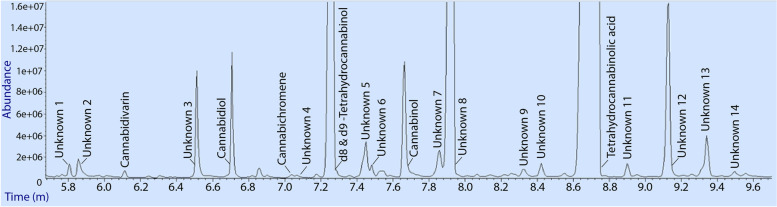
Total ion current chromatogram from a 3.5 ± 0.1 mg of trichomes from mixed inflorescences plant material with two different *Cannabis sativa* accessions (a chemotype I and a chemotype III). *Cannabis sativa* plant sample filtrate showing elution of 7 cannabinoids identified from standards and elution of 14 unknown compounds likely to represent unidentified cannabinoids

Retention times, quantification, and qualifying ions used for the analysis of cannabinoids and unknown compounds following extraction and derivatisation from a mixed flower plant material (flowers, young leaf, and trichomes) samples, are shown in Table 4. The same extraction and analytical method as described in the methodology section were applied to a smaller weight sample (3.5 ± 0.1 mg) of trichome extract. The total ion current of extraction performed on trichomes is shown in Fig. 6. Cannabinoid diversity was greatly increased in the trichome extraction used in this study. To obtain satisfactory peak shape and abundance, it was necessary to use a sufficient sample size that resulted in three dominant peaks being overloaded and unable to be quantified. These included a coeluting combination of Δ^8^-THC and Δ^9^-THC, THCA, and 14 unknowns (Fig. 6). The ability to analyse a greater number of unknown compounds that likely represent cannabinoids or components in synthetic cannabinoid pathways is greatly increased by applying an initial trichome extraction step. Retention times, quantification, and qualifying ions used for the analysis of cannabinoids and unknown compounds following extraction and derivatisation from a trichome sample from a mixed inflorescence accessions (the previous shown in Table 3) are shown in Table 4.

**Table 4:** Retention time, quantification and qualifying ions of cannabinoids and unknown compounds from 3.5 ± 0.1 mg trichome sample separated from the mixed inflorescences plant material with two different *Cannabis sativa* accessions (a chemotype I and a chemotype III)

Compound	Retention time (min)	Quantifier ion	Qualifier ions	Sample 1 (mg/kg)	Sample 2 (mg/kg)	Sample 3 (mg/kg)
Unknown 1	5.80	333	333, 292	–	–	–
Unknown 2	5.86	313	328, 269	–	–	–
Cannabidivarin	6.11	362	309, 273	229	294	244
Unknown 3	6.51	343	358, 315	–	–	–
Cannabidiol	6.71	390	337, 301	3006	3657	3362
Cannabichromene	7.04	303	246, 371	174	214	192
Unknown 4	7.07	318	303, 156	–	–	–
Δ^8^-tetrahydrocannabinol	7.24	386	303, 330	7933	9733	8541
Δ^9^-tetrahydrocannabinol	7.24	371	386, 315	7697	9431	8385
Unknown 5	7.45	303	318, 246	–	–	–
Unknown 6	7.49	474	391, 403	–	–	–
Cannabinol	7.66	367	382, 310	275	368	313
Unknown 7	7.86	391	433, 474	–	–	–
Unknown 8	7.91	459	491, 559	–	–	–
Unknown 9	8.32	455	367, 293	–	–	–
Unknown 10	8.43	575	447, 500	–	–	–
Tetrahydrocannabinolic acid	8.67	487	488, 502	149776	171757	179447
Unknown 11	8.90	503	413, 487	–	–	–
Unknown 12	9.12	483	395, 321	–	–	–
Unknown 13	9.34	419	156, 257	–	–	–
Unknown 14	9.49	501	519, 355	–	–	–

## Discussion

This study demonstrated a simple method of extraction and analysis of cannabinoids able to analyse and quantify 10 known cannabinoids and a further 14 compounds suggestive of cannabinoid chemistry based on GC-MS spectral profiles. The use of trichome separation enhanced the number of peaks representing cannabinoids, especially those present in low concentrations. As shown in Table 5, the extraction method presented here reduced the amount of chemicals and plant material required, decreased operator time from previous work, and allowed well separated and quantifiable peaks using GC-MS. Due to the low concentration of the non-abundant cannabinoids, GC-MS is an ideal analytical platform for cannabinoid analysis due to its high sensitivity (Leghissa et al. 2018b). Only the 10 cannabinoids from the certified standard mix could be identified with certainty. Compounds of low abundance were marked as unknown and identified as part of the cannabinoid group based on spectral profiles containing greater than two dominant fragmentation ions present in other known cannabinoids. Further development of this method will work toward a fully quantitative method, presented as recovery rate (%) using a known amount of standard spiked into a sample matrix. This method, in its current form, serves to provide semi-quantitative data and has been successfully employed to compare plant metabolic responses through cannabinoid analysis to various environmental challenges (De Prato et al. 2022a, b).

**Table 5:** Differences and improvements reported in the current method with respect to the most relevant and recent GC-MS studies for the separation, extraction, and cannabinoid analysis of *Cannabis sativa* inflorescence plant material

Typology	Reference cited	Previous operation used	Improvement	Differences
Separation of material	Nil	–	Separation of trichomes from inflorescence plant material through freeze treatment and sieving	Detection of low abundance cannabinoids
Plant material	Nil	–	3.5 ± 0.1 mg of trichomes material for extraction	Less plant material. Increased number of cannabinoids detection and avoiding GC-MS blockage
Plant material	(Cardenia et al. 2018)	50 mg of plant material	10.5 ± 0.3 mg of plant material	Less plant material
Chemicals and reagents	Cardenia et al. (2018)	Extraction of chloroform and methanol (1:9)	Extraction in methanol	Avoiding chloroform (toxic)
Chemicals and reagents	(Cardenia et al. 2018)	Extraction in 20 mL of solvent mix	Extraction in 1.5 mL of methanol	Less chemical used
Chemicals and reagents	(Cardenia et al. 2018)	Analysis from methanol	Analysis from n-Hexane	Easier for the operator and avoiding plant material filtering
Derivatisation	(Leghissa et al. 2018b)	Use of BSTFA	Comparison of BSTFA and MSTFA	Choice of MSTFA for better performance
Derivatisation	(Cardenia et al. 2018)	50 μL of pyridine and 150 μL of derivatisation agent	Only 90 μL derivatisation agent	Less agent saves costs for routine analysis

Cannabinoids, together with terpenes, are present in *C. sativa* plants throughout the plant cycle from the vegetative stage to seed ripening (Bócsa et al. 1997; Pacifico et al. 2008; Richins et al. 2018; Small et al. 2003). The concentration of these compounds tends to be higher in female plants, in younger flowers, and especially in the resin secreted by the trichomes (Casiraghi et al. 2018; Small and Naraine 2016; Small et al. 2003). Several studies have shown that the cannabinoid profile is affected by plant stress, nutrition, and environmental conditions (Coffman and Gentner 1975; Sikora et al. 2011; Small et al. 2003). The concentration of a single cannabinoid can change over a plant life cycle, and higher cannabinoid concentrations occur near flowers and bracts (Richins et al. 2018); this is where trichomes are situated (Casiraghi et al. 2018). Direct extraction from trichomes directly targets cannabinoids, avoiding unwanted metabolites and proteins. In the present study, trichomes were separated by freezing the plant material at – 4 °C for 30 min, and then gently shaking through a thin nylon mesh (40 μm). This allowed the brittle resin to be harvested, creating a clean cannabinoid-rich substance. This process led to improved detection of cannabinoids and reduced ion suppression at the analytical source. It should be noted that if the operator is interested solely in abundant cannabinoid analysis, such as THC and CBD only, then there is no need to extract trichomes; this step may be omitted, and, therefore, the total plant material should be used. If the aim is to analyse cannabinoids of low abundance or produce a cannabinoid-rich product devoid of the majority of plant material, this simple and effective method separates the trichomes from the remainder of the plant material. Removing plant material can increase the cannabinoid extraction fraction and reduce deleterious ion suppressive effects at the ion source of the mass spectrometer. This could be important in the medicinal field to identify minor metabolites and highlight their potential therapeutic effects.

Extraction of polyphenols and other compounds from plant material is a common practice in the food industry. However, different techniques are used, depending on the metabolite typology (Brglez Mojzer et al. 2016). Cannabinoids, as secondary metabolites of *C. sativa*, are terpenophenolic that can be grouped into terpenes and cannabinoids (Aizpurua-Olaizola et al. 2016). Terpenes are volatile and more delicate compounds that need to be extracted at low temperatures through hydro distilling (Ibrahim et al. 2019). Cannabinoids, however, have been extracted with solvents based on their polar/non-polar structure (Casiraghi et al. 2018). Indeed, for these bioactive compounds, a dynamic maceration by ethanol at room temperature has been demonstrated to be the easiest and cost-efficient (Citti et al. 2018; Pellati et al. 2018). For example, a mixture at 9:1 (v:v) of methanol and chloroform has been utilised by Cardenia et al. (2018) on hemp inflorescences. In the current study, chloroform utilisation was avoided as (a) it is hazardous to transport and store, and expensive; (b) too aggressive on the targeted chemicals, (c) it is harmful to operators with no extra benefit on extraction (Mudge et al. 2017; Pellati et al. 2018), and (d) when dealing with potential medicinal *Cannabis*, toxic solvents should be avoided (Citti et al. 2018). Also, cannabinoids can dissolve in non-polar compounds (Pellati et al. 2018). The method developed in the present study differs from previous ones because, after the first maceration in MeOH, purified water and n-Hexane were used for further extraction before centrifugation. This allowed the separation of the water and MeOH solution from the n-Hexane upper layer of the tube where cannabinoids were dissolved. Adding n-Hexane allows fewer sugar, chlorophylls, and polysaccharides in the extract than methanol (Citti et al. 2018), with a cleaner extraction of the cannabinoids and easing the operator operation on transfer to GC-MS vial. During this operation, possible contamination can be avoided while extracting only from the upper layer. The extraction ability of methanol and the non-polar filtering and lighter molecular weight characteristics of n-Hexane were utilised.

Over the last few years, GC, coupled with FID or MS has become popular for the identification and quantification of cannabinoids (Citti et al. 2018; Leghissa et al. 2018b; Nahar et al. 2020). Consequently, some government authorities have nominated GC-MS as the standard instrument (Casiraghi et al. 2018). MS has extremely high sensitivity and the options of using software and libraries for compound detection (Citti et al. 2018; Leghissa et al. 2018b). However, extra time for preparation by derivatisation is needed in comparison to HP-LC, which makes GC-MS unattractive for commercial routine analysis. Recent developments have improved these processes by using nitrogen or hydrogen as the carrier gas, together with higher gas flow, shorter and narrower columns, and shorter oven temperature increases (Nahar et al. 2020). FID has been used to quantify cannabinoids and terpenoids rapidly (Ibrahim et al. 2018; Nahar et al. 2020) but lacks in the ability to detect novel compounds and quantify coeluting compounds. Coupling GC to MS, whilst requiring higher operator skill and expense, provides the ability to detect untargeted molecules, which is a great advantage for metabolomics studies and to correlate primary metabolites and cannabinoids (Capriotti et al. 2021; Rashid et al. 2021), as shown in the present study. Utilisation of MS through metabolomics analysis can drive breeding programs (Bueno and Lopes 2020), which are presently highly sought in the *Cannabis* industry (Cosentino et al. 2012; Hall et al. 2012; Naim-Feil et al. 2021). In the present study, high flow helium and a fast oven ramping temperatures allowed our instrument run time to be under 15 min. Derivatisation is required to analyse THCA and CBDA by GCMS (Fodor and Molnár-Perl 2017) as cannabinoids in acidic form (i.e. THCA and CBDA) will experience decarboxylation at high temperatures. The efficacy of the derivatisation agents MSTFA-TMS and BSTFA-TMS were assessed during the method development of this study (data not shown), and we found less artefact and enhanced peak shape using MSTFA-TMS.

Most cannabinoids are present only in low quantities, their pharmacological effects have not been studied yet, and many potential compounds remain untargeted (Capriotti et al. 2021; Carvalho et al. 2017). For example, novel and important cannabinoids with psychoactive properties, such as Δ^9^-Tetrahydrocannabiphorol, were recently discovered through high resolution MS (Citti et al. 2019). Currently, for commercially available standards, only 11 cannabinoids of interest are synthesised for routine analysis (Carvalho et al. 2017), which makes it more difficult to identify low abundance cannabinoids. The method developed in the present study showed that the direct extraction from trichomes allowed the detection of a range of low abundance and untargeted cannabinoids, which could potentially have therapeutic properties. Further research is needed to determine the therapeutical implications of cannabinoids other than THC and CBD and this method highlights the abundance of a much greater range of commonly present cannabinoids in hemp. By using this method and semi-quantitative analysis, it would be possible to use principal component analysis studies tied with clinical trials to elucidate cannabinoids that can have medical significance other than the commonly studied THC and CBD.

## Conclusions

The preliminary step of separating trichomes from plant material led to a greater amount of cannabinoids being detected than from raw plant material. The amount of plant material required and reagent required was greatly reduced from previous studies. Despite method development, two peaks coeluted, and the power of the mass spectrometer was required for their quantification. Further method development could involve oven ramping modifications or the use of a wax-based column to obtain separation of all standards. This would allow a transition to the use of an alternate and cheaper detection tool, such as FID. All compounds detected in this study had a unique retention time and quantifier and qualifier ion combination that could be used for identification and quantification. The future of *C. sativa* as a medicinal source of cannabinoids will involve the detection of individual low abundance cannabinoids and clinical trials using combinations of these cannabinoids. Future research will involve elucidating complete metabolic profile and synthesis steps towards the formation of secondary plant metabolites, such as cannabinoids.

## Limitations

In Australia, the current legislation includes any substance with trace of THC under a ‘Schedule 8: Controlled Drug’ and the use, transformation and handling is under a permit and strictly regulated by law. Acquiring, storing and handling a cannabinoids standard is included in the abovementioned schedule which made fairly difficult acquiring and processing the results from this study. For this reason, we were be not able to acquire extra cannabinoid standard and research further on the cannabinoids present on our samples, even for research purpose.

## Data Availability

The datasets used and/or analysed during the current study are available from the corresponding author on reasonable request.

## Abbreviations

BSTFA: N,O-bis(trimethylsilyl)trifluoroacetamide
CB_1_: Endocannabinoid system 1
CB_2_: Endocannabinoid system 2
CBC: Cannabichromene
CBD: Cannabidiol
CBDA: Cannabidiolic acid
CBDV: Cannabidivarin
CBG: Cannabigerol
CBGA: Cannabigerolic acid
CBN: Cannabinol
EI: Electron ionisation
FID: Flame ionisation detector
GC: Gas chromatography
HPLC: High-performance liquid chromatography
MS: Mass spectrometry
MSD: Mass selective detector
MSTFA: N-methyl-N-(trimethylsilyl)trifluoroacetamide
THCA: Delta-9-tetrahydrocannabinolic acid
THCV: Delta-9-tetrahydrocannabivarin
THCVA: Tetrahydrocannabivarin acid
TMCS: Chlorotrimethylsilane
TRPV1: Transient receptor potential cation channel subfamily V member 1
UV: Ultraviolet detector
Δ^8^-THC: Delta-8-tetrahydrocannabinol
Δ^9^-THC or THC: Delta-9-tetrahydrocannabinol
Δ^9^-THCP: Delta-9-tetrahydrocannabiphorol
